# Cerebro-Spinal Fluid Escaping from the Nose

**Published:** 1898-11-12

**Authors:** 


### CEREBRO-SPINAL FLUID ESCAPING FROM THE NOSE.

The very curious and interesting case shown by Dr. St. Clair Thomson at the Royal Medical and Chirurgical Society on Tuesday last must be looked on as one of the curiosities of medicine. Nevertheless, it is well to beware of such things, partly lest one might be led to give an erroneous opinion in any such case as might come before one, but especially because it is just possible that a slighter degree of the same sort of lesion as was the cause of the leak of cerebro-spinal fluid in Dr. Thomson's case may in other cases be the unsuspected origin of cerebral complications. In a large proportion of the reported cases of this affection which Dr. Thomson has been able to collect, cerebral symptoms were associated with this cerebro-spinal rhinorrhœa, and, indeed, in the cases in which there is a record of a fatal termination this was in every instance due to cerebral complications, and it is not difficult to see that an intermittently flowing fistula might be even more likely to communicate septic infection to the sub-arachnoid space even than one in which the outflowing stream was practically continuous, as in the cases described. Summarised briefly, the condition in the case brought forward by Dr. St. Clair Thomson was as follows: A young woman, in apparently good health, and without any tangible cause, commences to suffer from the escape of cerebro-spinal fluid from the nose, which, however, produces no other symptom than the mechanical one of inconvenience. At first the flow was intermittent in its appearance, but for the last three years, with the exception of three intermissions, it has been continuous day and night. On two occasions the cessation continued for one month, and on the other for two months. To all intents and purposes the patient remains in perfect health; in fact, in one respect she is better than she was before the flow was established, for she is now free from severe headaches to which she used to be subject.

Similar cases have already been published, but in a majority of instances a true diagnosis does not appear to have been arrived at, the dropping of fluid from the nose being ascribed to hypersecretion from the mucous membrane of the nasal cavities or its accessory cavities.

What seems to be clear is that the sub-arachnoid fluid can escape through the nose without trauma or new growth to explain how it effects an exit. This condition is an affection of adult life, the age incidence falling between the extremes of 18 and 65. The flow in most cases commences gradually; it occurs in drops, much as the flow of blood in epistaxis, and in carefully recorded cases it is continuous both by day and by night, although in the latter period it may be swallowed during sleep. Generally it makes its escape from one nostril only, in the majority of instances from the left. The fluid which escapes amounts to about half a litre in the twenty-four hours; it is as clear and limpid as spring water, perfectly free from odour and taste, or is only slightly salt; and, curiously, seeing the passage down which it has to flow before it can be collected, if it is collected with care it is found to be absolutely sterile.

In a large number of cases there were cerebral complications, and in a certain number of instances ocular affections were discovered. The affection has been known to last for five and twelve years, while spontaneous intermission and even complete cessation sometimes occur. The diagnosis, which of course has to be made from hypersecretion from the lining of the nose and its accessory cavities, has to be arrived at by a careful consideration of the character of the fluid.

As to treatment, the important point would seem to be to abstain from all intra-nasal medication, so as to avoid risk of infecting the secretion. The fact is that, until we know more than we do about the pathology of the affection, it is desirable to refrain from any direct attempt to check the flow.

